# Comparison of Bone Mineral Density in Thalassemia Major Patients with Healthy Controls

**DOI:** 10.1155/2015/648349

**Published:** 2015-12-31

**Authors:** Mahesh Chand Meena, Alok Hemal, Mukul Satija, Shilpa Khanna Arora, Shahina Bano

**Affiliations:** ^1^Department of Pediatrics, PGIMER and Dr. RML Hospital, New Delhi 110001, India; ^2^Department of Radiology, PGIMER and Dr. RML Hospital, New Delhi 110001, India

## Abstract

Chronic hemoglobinopathies like thalassemia are associated with many osteopathies like osteoporosis.* Methods*. This observational study was carried out to compare the bone mineral density (BMD) in transfusion dependent thalassemics with that of healthy controls. Thirty-two thalassemia patients, aged 2–18 years, and 32 age and sex matched controls were studied. The bone mineral concentration (BMC) and BMD were assessed at lumbar spine, distal radius, and neck of femur. Biochemical parameters like serum calcium and vitamin D levels were also assessed.* Results*. The BMC of neck of femur was significantly low in cases in comparison to controls. We also observed significantly lower BMD at the lumbar spine in cases in comparison to controls. A significantly positive correlation was observed between serum calcium levels and BMD at neck of femur.* Conclusion*. Hence, low serum calcium may be used as a predictor of low BMD especially in populations where incidence of hypovitaminosis D is very high.

## 1. Introduction

The management of patients with thalassemia has improved markedly over the past few decades with the use of optimized transfusion programs and chelating therapy. There has been a considerable improvement in the life expectancy and the quality of life of these patients. With prolongation in the life expectancy, it has been observed that this hemoglobinopathy is associated with a variety of bone disorders like deformities, bone pains, delayed bone age, growth failure, rickets, scoliosis, spinal deformities, nerve compression, pathologic fractures, osteopenia, and osteoporosis [1]. Apart from disease process per se, high-dose iron chelating therapy with desferrioxamine may also contribute to osteopenia and osteoporosis [2, 3].

Osteoporosis is a significant cause of morbidity in these patients [4]. It is characterized by low bone mass and disruption of bone architecture, resulting in reduced bone strength and increased risk of fractures [5, 6]. Though many techniques are available for quantitative assessment of the degree of osteoporosis and total bone mass, bone density measurement by Dual Energy X-Ray Absorptiometry (DEXA) of lumbar spine, femoral neck, and distal radius is considered a very reliable and noninvasive technique [7].

This study was carried out to evaluate the bone mineral density (BMD) in children with thalassemia major by DEXA and to compare with healthy controls.

## 2. Methods

This cross-sectional observational study was conducted in a tertiary care center in Delhi from November 2011 to January 2013. Clearance was taken from the institutional ethical committee. The primary objective of the study was to compare the BMD of thalassemia major patients with that of age and sex matched controls. The sample size was calculated considering a power of 80% using a 2-sided Student's *t*-test and *α*-error = 5%. Assuming mean BMD to be 0.63 ± 0.076 g/cm^2^ in patients and 0.78 ± 0.3 g/cm^2^ in controls, the sample size was calculated to be 32 in each group.

Thalassemia patients, aged 2–18 years, attending the thalassemia clinic of the hospital, were enrolled consecutively. The patients who were taking medicines like antiepileptic drugs, oral calcium, vitamin D, and so forth that affect bone mineral density were excluded. A total of 32 patients and 32 age and sex matched healthy controls were recruited after an informed written consent was obtained from the parents. Assent was taken from children older than 7 years.

The details of all the subjects were recorded including age, sex, weight, height, and body mass index (BMI). In addition, details like duration of disease, blood transfusion, chelation, and other treatment histories were recorded for the patients. The biochemical assessment included determination of serum calcium, phosphorus, and 25-hydroxy vitamin D levels.

All the subjects underwent DEXA scan using HOLOGIC (Discovery QDR series) bone densitometer. Bone Mineral Content (BMC), areal bone mineral density (aBMD), and volumetric bone mineral density (vBMD) were obtained at lumbar spine, femoral neck, and distal radius. BMC was expressed in gram, aBMD in gram/cm^2^, and volumetric BMD in gram/cm^3^. The “*T* score” and the “*Z* score” were also calculated at these 3 sites after DEXA scan. The *T* score refers to the number of standard deviations above or below the mean for a healthy 30-year-old adult of the same sex and ethnicity as the patient. A *T* score of −1.0 or higher is considered normal. Osteopenia has been defined as a “*T* score” between −1.0 and −2.5 while osteoporosis has been defined as score of  −2.5 or lower.  *T* score does not have clinical utility in children and adolescents. *Z* score is the preferred parameter in children which is calculated as the number of standard deviations above or below the mean for the patient's age, sex, and ethnicity. The diagnosis of osteoporosis and osteopenia in childhood is not made on densitometric data alone especially in absence of a clinically significant fracture history.

Statistical analysis was performed by the SPSS program for Windows, version 17.0. Normally distributed continuous variables were compared using the unpaired *t*-test, whereas the Mann-Whitney *U* test was used for those variables that were not normally distributed. Categorical variables were analyzed using either the chi square test or Fisher's exact test. Spearman's correlation was also performed between BMD, age, sex, body mass index (BMI), chelation, ferritin, calcium, and vitamin D in cases and controls. *P* < 0.05 was considered statistically significant.

## 3. Results

A total of 32 thalassemic patients (21 male, 11 female) between 5 to 16 years of age (mean ± SD = 10.03 ± 3.03) were studied along with 32 age and sex matched controls. The mean height (133.84 ± 13.40 cm), weight (32.28 ± 10.25 kg), and BMI (17.70 ± 3.86 kg/m^2^) of cases were lower in comparison to controls but only weight (*P* = 0.023) and BMI (*P* = 0.030) were statistically significant. The mean age of receiving first blood transfusion in the patients was 3.72 ± 1.14 years (range = 2–6 years). The mean serum ferritin value of the thalassemia patients was 3349 ± 2012.9 ng/mL (range = 1082–9002 ng/mL). The mean value of total serum iron in children was 459.13 ± 119.47 *µ*g/dL (range = 320–765 *µ*g/dL).

Comparison of biochemical profile in the two groups revealed a significantly lower mean serum calcium level in cases (8.69 ± 1.04 mg/dL) (range = 7–10.4 mg/dL) in comparison to the controls (9.44 ± 0.61 mg/dL) (range = 8.3–10.8 mg/dL) (*P* = 0.001). The mean serum phosphorus levels were 3.97 ± 0.53 mg/dL (range = 3.1–4.9 mg/dL) in cases and 3.73 ± 0.60 mg/dl (range = 2.5–4.9 mg/dL) in controls and the difference was not statistically significant (*P* = 0.094). The mean serum alkaline phosphatase values were 241.54 ± 89.30 IU/L (range = 112–432 IU/L) in cases and 287 ± 101.94 IU/L (range = 112–607 IU/L) in controls and it was not statistically significant (*P* = 0.056). The mean levels of vitamin D were 19.74 ± 10.71 nmol/L (range = 12–63.4 nmol/L) in cases and 23.21 ± 11.10 nmol/L (range = 11.4–63.4 nmol/L) in controls and the difference was not statistically significant (*P* = 0.144). Analysis of serum 25-hydroxy vitamin D demonstrated that none of the subjects in both groups had levels in the normal range (>75 nmol/L) and there was no significant difference in the mean values between the two. Majority (59.4%) of the cases demonstrated moderate 25-OH Vit D deficiency (12.5 to 25 nmol/L), 21.9% were severely deficient (<12.5 nmol/L), and the rest had levels in the minor deficiency (25–50 nmol/L) or insufficient range (50–75 nmol/L).

The BMC and BMD were assessed at lumbar spine, distal radius, and neck of femur and the values are depicted in Table 1. The BMC of neck of femur was significantly low in cases (1.87 ± 0.51 gm) in comparison to controls (14.13 ± 12.23 gm) (*P* < 0.001). The mean BMC at lumbar spine (LS) as well as distal radius (DR) was also lower for the cases (LS, 20.55 ± 8.22 gm; DR, 3.711 ± 2.24 gm) in comparison to controls (LS, 20.98 ± 6.48 gm; DR, 4.60 ± 2.68 gm) but the difference was not statistically significant (LS, *P* = 0.818; DR, *P* = 0.143). The mean BMD for thalassemia cases (0.48 ± 0.17 gm/cm^3^) at lumbar spine was significantly low in comparison to controls (0.568 ± 0.110 gm/cm^3^) (*P* = 0.013) but the difference was not statistically significant at distal radius (*P* = 0.933) and neck of femur (*P* = 0.495). The difference in mean BMD at lumbar spine was more marked in children aged more than 10 years.

Correlation of bone mineral density at all three sites with calcium and other parameters was done for the patients as depicted in Table 2. A positive correlation was found between calcium level and BMD at all three sites, namely, lumbar spine (*r* = 0.116, *P* = 0.526), distal radius (*r* = 0.063, *P* = 0.733), and neck of femur (*r* = 0.392, *P* = 0.026). However, the difference was statistically significant only at neck of femur. A positive correlation was also observed between vitamin D level and BMD at all the three sites but the difference was not statistically significant (Table 2).

Bone mineral density at all the sites showed a negative correlation with age although the difference was not statistically significant as depicted in Table 2. No significant correlation was seen for BMI, serum ferritin, and chelation with the BMD at any of the sites. There was no significant difference between BMD of male and BMD of female thalassemia patients.

## 4. Discussion

During the last decade, osteopenia or osteoporosis has been reported in approximately 40–50% of well treated thalassemia major patients in different studies and is major cause of morbidity in these patients [8, 9]. Bone changes in thalassemic patients are due to increased marrow erythropoiesis and extensive iron deposition resulting in expansion of bone marrow cavities and reduced trabecular bone volume, leading to decreased bone tissue and osteoporosis [4, 10]. Chelation is another important risk factor for causing osteoporosis in these patients as high dose desferrioxamine therapy causes decrease in the differentiation and proliferation of bone-forming cells, decreases collagen formation, and increases osteoblast programmed cell death. Chelation also leads to deficiency of vitamins and minerals like vitamin D and zinc, which in turn worsen the bone health [2, 3]. Presence of other endocrinopathies like hypothyroidism, hypoparathyroidism, diabetes mellitus, and hypogonadism also contributes to bone disease.

The present study observed significantly lower BMD at the lumbar spine which is in accordance with previous studies suggesting that the lumbar spine is more affected in thalassemia patients in comparison to the other sites of BMD assessment [5].

In this study, the mean weight and body mass index of cases were significantly lower than controls. The mean height of cases was also lower though the difference was not statistically significant. These changes can be explained due to chronic illness and endocrinal changes due to iron overload.

Vitamin D deficiency was observed in almost all the cases of thalassemia in the present study. The main causes of vitamin D deficiency in thalassemic patients are nutritional deficiency and defective hydroxylation of vitamin D in liver due to hemochromatosis. But there was no significant difference in vitamin D levels between cases and controls. This could be due to vitamin D deficiency being a common finding in the general population in India.

Hypocalcemia is a common finding in thalassemic patients. In our study, serum calcium level <8 mg/dL was found in 31.3% of the cases, while none in the control group had serum calcium value <8 mg/dL. There was a statistically significant difference in serum calcium value between cases and controls. This suggests that factors other than vitamin D deficiency also play a role in causing hypocalcemia in thalassemics.

In this study, there was a significant difference in bone mineral density at lumbar spine between cases and controls (*P* = 0.013) but at distal radius and neck of femur in cases and controls it was not statistically significant (*P* = 0.933). It is likely that the differences in the BMD of cases and controls would have been more striking if the control population had normal serum Vit D levels. This is a limitation of the present study that the majority of the control group too had vitamin D deficiency. We also observed that the bone mineral density was most affected at lumbar spine which is in accordance with a previous study by Jensen et al. [7].

Bone mineral density at all the sites showed a negative correlation, though not statistically significant, with age. This suggests that BMD decreases with advancing age, as previously observed in a study of Indian thalassemia children between 10 and 25 years of age [6]. We also observed a positive correlation between BMD and vitamin D levels but it was not statistically significant. Similarly, positive correlation was found between calcium level and BMD at all three sites but the difference was statistically significant only at neck of femur. A statistically significant positive correlation might have become evident at other sites as well if a larger number of cases were evaluated in this study. Thus, the present study suggests that not only low serum vitamin D levels but also low serum calcium levels can be an important predictor of low bone mineral density in thalassemics.

Thalassemia International Federation [11] suggests annual BMD assessment in these patients, starting in adolescence. The availability and affordability of DEXA are a major limitation in many poor as well as developing nations. This study observed that serum calcium and vitamin D deficiency and low BMD are widely present in thalassemic children. Low serum calcium may be used as predictor for low BMD, more so in populations where incidence of hypovitaminosis D is very high. Thus, in regions with poor availability of DEXA, serum calcium and vitamin D levels must be routinely monitored in thalassemics especially after the age of 10 for evaluation of bone health.

## Figures and Tables

**Table 1:** Bone mineral content (BMC) and bone mineral density (BMD) in cases and control at lumbar spine (LS), distal radius (DR), and neck of femur (NF).

	Controls		Cases		*P* value
	Mean ± SD	Min–max	Mean ± SD	Min–max	
BMC_LS (gm)	20.98 ± 6.48	13.28–38.18	20.55 ± 8.22	13.28–38.18	0.818
BMD_LS (gm/cm^3^)	0.568 ± 0.110	0.379–0.821	0.48 ± 0.17	0.211–0.791	0.013
BMC_DR (gm)	4.60 ± 2.68	0.60–10.01	3.711 ± 2.24	1.06–10.81	0.143
BMD_DR (gm/cm^3^)	0.323 ± 0.06	0.218–0.441	0.324 ± 0.99	0.110–0.560	0.933
BMC_NF (gm)	14.13 ± 12.23	1.2–37.3	1.87 ± 0.51	1.2–2.9	<0.001
BMD_NF (gm/cm^3^)	0.496 ± 0.17	0.321–0.869	0.464 ± 0.198	0.230–0.907	0.495

**Table 2:** Correlation of bone mineral density at lumbar spine (LS), distal radius (DR), and neck of femur (NF) in cases.

		BMD_LS	BMD_DR	BMD_NF
Age	*r*	−0.052	−0.289	−0.066
	*P* value	0.776	0.109	0.718

Sex	*r*	−0.078	0.011	−0.153
	*P* value	0.670	0.954	0.402

BMI	*r*	0.393	0.003	0.039
	*P* value	0.026	0.987	0.832

S. ferritin	*r*	0.201	0.043	0.180
	*P* value	0.270	0.817	0.325

Chelation	*r*	−0.065	−0.208	−0.064
	*P* value	0.726	0.254	0.728

Ca	*r*	0.116	0.063	0.392
	*P* value	0.526	0.733	0.026

Vit D levels	*r*	0.086	0.236	0.236
	*P* value	0.641	0.193	0.194
